# (6*S*)-2,4-Di-*tert*-butyl-6-[(4*S*,5*R*)-3-iso­propyl-4-methyl-5-phenyloxazolidin-2-yl]phenol

**DOI:** 10.1107/S1600536810009074

**Published:** 2010-03-24

**Authors:** Ian Sean Campbell, Kate L. Edler, Raleigh W. Parrott, Shawn R. Hitchcock, Gregory M. Ferrence

**Affiliations:** a CB 4160, Department of Chemistry, Illinois State University, Normal, IL 61790, USA

## Abstract

The title oxazolidine compound, C_27_H_39_NO_2_, was synthesized from *N*-isopropyl­norephedrine. The dihedral angle between the aromatic rings is 70.33 (5)°. The N atom of the heterocycle is oriented to allow intra­molecular O—H⋯N hydrogen bonding with the hydr­oxy substituent.

## Related literature

For related structures and background to chiral oxazolidines, see: Agami & Couty (2004 ▶); Anderson *et al.* (2010 ▶); Bourne *et al.* (1997 ▶); Duffy *et al.* (2004 ▶); Hitchcock *et al.* (2004 ▶); Koyanagi *et al.* (2010 ▶); Parrott & Hitchcock (2007 ▶); Parrott *et al.* (2008 ▶). The synthesis and absolute configuration assignment of the title compound is described by Parrott *et al.* (2008 ▶). The absolute configuration assignment is based on both optical activity measurements and on the known stereochemistry of the commercially obtained optically pure norephedrine from which it was prepared (Parrott *et al.*, 2008 ▶). For geometry checks using *Mogul*, see: Bruno *et al.* (2004 ▶). For ring puckering analysis, see: Boeyens (1978 ▶); Cremer & Pople (1975 ▶); Spek (2009 ▶). For a description of the *Jmol* toolkit for the preparation of enhanced figures, see: McMahon & Hanson (2008).
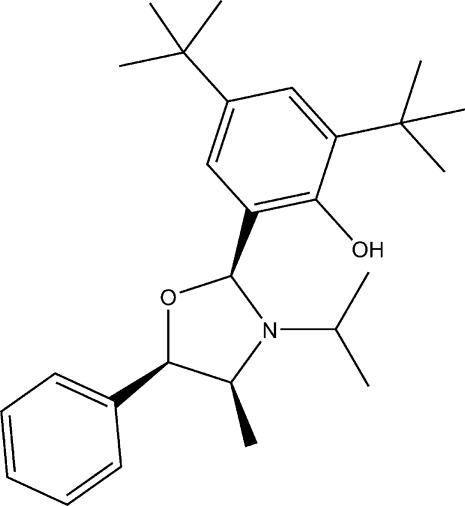


## Experimental

### 

#### Crystal data

C_27_H_39_NO_2_
*M* *_r_* = 409.59
Monoclinic, 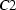

*a* = 18.9564 (19) Å
*b* = 6.9943 (7) Å
*c* = 18.3388 (19) Å
β = 91.833 (2)°
*V* = 2430.2 (4) Å^3^
*Z* = 4
Mo *K*α radiation
μ = 0.07 mm^−1^
*T* = 140 K
0.55 × 0.27 × 0.11 mm

#### Data collection

Bruker SMART APEX CCD diffractometer
Absorption correction: multi-scan (*SADABS*; Bruker, 2008 ▶) *T*_min_ = 0.809, *T*_max_ = 0.992
14330 measured reflections
3938 independent reflections
3568 reflections with *I* > 2σ(*I*)
*R*_int_ = 0.035

#### Refinement

*R*[*F* ^2^ > 2σ(*F* ^2^)] = 0.038
*wR*(*F* ^2^) = 0.097
*S* = 1.06
3938 reflections
275 parameters
1 restraint
H atoms treated by a mixture of independent and constrained refinement
Δρ_max_ = 0.31 e Å^−3^
Δρ_min_ = −0.21 e Å^−3^

### 

Data collection: *APEX2* (Bruker, 2008 ▶); cell refinement: *APEX2* and *SAINT* (Bruker, 2008 ▶); data reduction: *SAINT*; program(s) used to solve structure: *SIR2004* (Burla *et al.*, 2005 ▶); program(s) used to refine structure: *SHELXL97* (Sheldrick, 2008 ▶); molecular graphics: *ORTEP-3* for Windows (Farrugia, 1997 ▶) and *Mercury* (Macrae *et al.*, 2008 ▶); software used to prepare material for publication: *WinGX* (Farrugia, 1999 ▶) and *publCIF* (McMahon & Westrip, 2008 ▶).

## Supplementary Material

Crystal structure: contains datablocks global, I. DOI: 10.1107/S1600536810009074/sj2720sup1.cif

Structure factors: contains datablocks I. DOI: 10.1107/S1600536810009074/sj2720Isup2.hkl

Additional supplementary materials:  crystallographic information; 3D view; checkCIF report

**Fig. 2.**
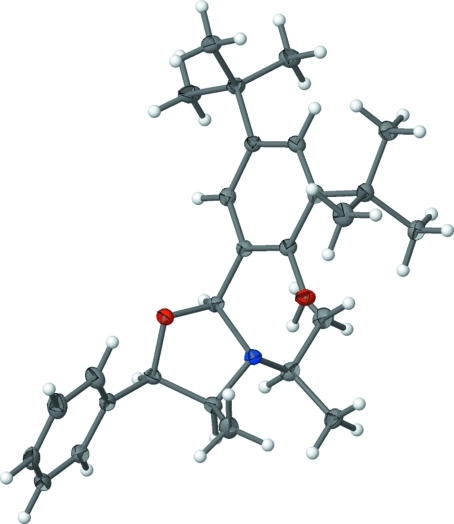
The enhanced Jmol figure of the title compound. This is the second in a series of three Jmol figures intended to illustrate some versatility of the program. In this Jmol, all interactive features are defined by using the graphical interface. In addition, the view associated with each script is changed to highlight the script contents. Some script artifacts occur and can only be remedied by hand-editing the scripts.

Enhanced figure: interactive version of Fig. 2

## Figures and Tables

**Table 1:** Hydrogen-bond geometry (Å, °)

*D*—H⋯*A*	*D*—H	H⋯*A*	*D*⋯*A*	*D*—H⋯*A*
O22—H22⋯N3	0.91 (3)	1.75 (2)	2.6180 (17)	158 (2)

**Fig. 1.**
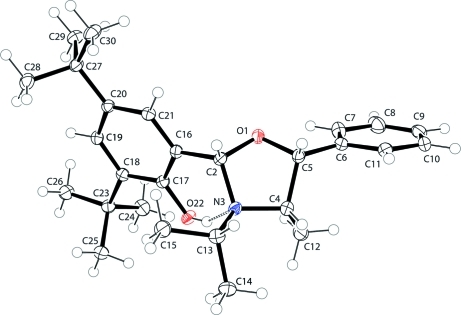
The molecular structure of the title compound with the atomic numbering scheme. Displacement ellipsoids are drawn at the 50% probability level. The intramolecular H-bonding is denoted by a dashed line.

## supplementary crystallographic information

### Comment

Chiral oxazolidines are useful templates for conducting asymmetric syntheses (Agami & Couty, 2004). In order to explore the utility of these compounds in the catalytic asymmetric addition of diethylzinc to aldehydes, we prepared a series of oxazolidines from (1R,2S)-ephedrine (Parrott & Hitchcock, 2007), and (1R,2S)-norephedrine (Parrott *et al.*, 2008). In the course of synthesizing these oxazolidines, we were able to obtain crystals suitable for X-ray crystallographic analysis.

Comparison of the title compound to the CSD structure refcode ROBWIO (Bourne *et al.*, 1997) shows similar bond lengths and angles. Differences in the two structures occur in the presence of two t-butyl groups on the phenyl ring (C18 and C20 in the title compound) and an isopropyl group on the nitrogen (N3 in the title compound) instead of a methyl group. A *Mogul* geometry check (Bruno *et al.*, 2004) indicates two angles to be unusual in both structures. Angle C2—N3—C4 in the title compound and the corresponding angle in ROBWIO are similar (106.2 (1) ° and 106.3 (2) ° respectively). However, angle C5—O1—C2 in the title compound is considered unusual (103.3 (1) °) while that of ROBWIO is not (110.8 (2) °). The difference could be due to ring compression from the additional steric bulk of the two t-butyl substituents on the phenyl ring present in the title compound. The distance between the hydrogen donor and acceptor (O22 and N3 in the title compound and their analogous partners) are indistinguishable at 2.6180 (17) Å and 2.638 (432) Å, respectively.

Ring puckering analysis using *PLATON* (Spek, 2009; Cremer & Pople, 1975; Boeyens, 1978) indicates Φ = -7.05 (19)° for the O1—C2—N3—C4—C5 ring, which is consistent with a formal conformational assignment in between an idealized ^1^E envelope and a ^1^T_5_ twist with O1 being the flap apex and C5 having a slight twist. The *anti*-relationship between the substituents on N3 and C2, C4, and C5 is necessary to support the intramolecular hydrogen bonding present.

About the Jmol enhanced figure:

The procedure for recreating the Jmol figure is provided in the hope that readers will find it useful for creating their own. We are reporting three related structures containing Jmol enhanced figures, one in this paper and the other two in other papers in this *Journal* (Anderson *et al.*, 2010; Koyanagi *et al.*, 2010).  The Jmol enhanced figures were created to illustrate a range of author convenience versus end user experience, ranging from a purely GUI driven experience for the author resulting in a less functional figure for the end user to a more sophisticated use of the Jmol scripting by the author resulting in a more polished and versatile figure for the end user. The buttons, check boxes and radio buttons in the three examples visually appear to be identical; however, the underlying code they execute results in significantly different overall responses by the Jmol visualizer.

By strictly authoring with the Jmol toolkit GUI, without text editing any code, generation of the figure is relatively quick and easy. However, doing so results in a final figure which has some significant limitations. In particular, when the end user manipulates the figure by, for example, a rotation, subsequent clicking of a radiobutton will result in the figure reseting to appear exactly as it appeared when the author saved the script. This includes all settings such as orientation and any other highlighting. This is the scenario illustrated by the Jmol enhanced figure associated with this Acta E article. The enhanced figure options were intentionally selected with an alteration of the structure's orientation, so that the molecule's orientation changes upon each option selected by the end user, which serves to emphasize the view that best show cases the selected option.

The Jmol options were created as follows:

Labels were added to atoms by navigating to the "label" sub-tab under the "select/label" tab and by checking the button "atom name" before turning the labels "on". The script was imported into a checkbox by navigating to the "checkbox" sub-tab under the "script" tab, and by clicking "import view".

The thermal displacement coloring was achieved by navigating to the "model" tab and by selecting "atomic displacement" next to the "colour" heading.

The color of particular atoms was changed by first selecting them. The atoms were selected by navigating to the "select/label" tab, turning the "highlight selection" on, and picking "within area" under "selection mode". The color of the atoms was changed by navigating to the "atoms" sub-tab and picking a color from the drop down box next to the "colour" heading.

The various atom styles were selected by navigating to the "model" tab and by selecting the atom style of choice next to the "overall style" heading.

The hydrogen bond was displayed by navigating to the "measurements" sub-tab under the "select/label" tab. The "distance" option next to the "mode" heading was then selected, followed by the hydrogen and acceptor atoms.

### Experimental

The title compound was synthesized as previously reported (Parrott *et al.*, 2008). Single crystals were grown by vapor diffusion of hexane into an ethyl acetate solution of the title compound.

### Refinement

All non-H atoms were refined anisotropically without disorder. All H atoms were initially identified through difference Fourier syntheses then, except for the O–H hydrogen atom, removed and included in the refinement in the riding-model approximation (C–H = 0.95, 0.98, and 1.00 Å for Ar–H, CH_3_ and CH; Uiso(H) = 1.2Ueq(C) except for methyl groups, where Uiso(H) = 1.5Ueq(C)). The OH H atom was freely refined isotropically. In the absence of significant anomalous scattering effects, Friedel pairs were merged.

### Crystal data

**Table:**

C_27_H_39_NO_2_	*F*(000) = 896
*M**_r_* = 409.59	*D*_x_ = 1.119 Mg m^−^^3^
Monoclinic, *C*2	Mo *K*α radiation, λ = 0.71073 Å
Hall symbol: C 2y	Cell parameters from 4163 reflections
*a* = 18.9564 (19) Å	θ = 2.4–30.0°
*b* = 6.9943 (7) Å	µ = 0.07 mm^−^^1^
*c* = 18.3388 (19) Å	*T* = 140 K
β = 91.833 (2)°	Prism, colourless
*V* = 2430.2 (4) Å^3^	0.55 × 0.27 × 0.11 mm
*Z* = 4	

### Data collection

**Table:**

Bruker SMART APEX CCD diffractometer	3938 independent reflections
Radiation source: sealed tube	3568 reflections with *I* > 2σ(*I*)
graphite	*R*_int_ = 0.035
ω scans	θ_max_ = 30.5°, θ_min_ = 2.2°
Absorption correction: multi-scan (*SADABS*; Bruker, 2008)	*h* = −26→26
*T*_min_ = 0.809, *T*_max_ = 0.992	*k* = −9→9
14330 measured reflections	*l* = −25→26

### Refinement

**Table:**

Refinement on *F*^2^	1 restraint
Least-squares matrix: full	H atoms treated by a mixture of independent and constrained refinement
*R*[*F*^2^ > 2σ(*F*^2^)] = 0.038	*w* = 1/[σ^2^(*F*_o_^2^) + (0.0531*P*)^2^ + 0.4486*P*] where *P* = (*F*_o_^2^ + 2*F*_c_^2^)/3
*wR*(*F*^2^) = 0.097	(Δ/σ)_max_ < 0.001
*S* = 1.06	Δρ_max_ = 0.31 e Å^−^^3^
3938 reflections	Δρ_min_ = −0.21 e Å^−^^3^
275 parameters	

### Special details

**Table:**

Geometry. All esds (except the esd in the dihedral angle between two l.s. planes) are estimated using the full covariance matrix. The cell esds are taken into account individually in the estimation of esds in distances, angles and torsion angles; correlations between esds in cell parameters are only used when they are defined by crystal symmetry. An approximate (isotropic) treatment of cell esds is used for estimating esds involving l.s. planes.

### Fractional atomic coordinates and isotropic or equivalent isotropic displacement parameters (Å^2^)

**Table:**

	*x*	*y*	*z*	*U*_iso_*/*U*_eq_	
O1	0.21602 (6)	0.71916 (17)	0.60579 (5)	0.0173 (2)	
C2	0.23499 (7)	0.5793 (2)	0.66002 (8)	0.0151 (3)	
H2	0.2784	0.5104	0.6451	0.018*	
N3	0.17413 (6)	0.4434 (2)	0.65832 (7)	0.0156 (2)	
C4	0.13878 (8)	0.4652 (3)	0.58521 (8)	0.0184 (3)	
H4	0.1378	0.3396	0.5591	0.022*	
C5	0.18794 (8)	0.6063 (2)	0.54634 (8)	0.0168 (3)	
H5	0.2272	0.5331	0.5242	0.02*	
C6	0.15197 (8)	0.7273 (3)	0.48843 (8)	0.0190 (3)	
C7	0.13772 (9)	0.6460 (3)	0.41968 (9)	0.0247 (4)	
H7	0.1551	0.5222	0.4087	0.03*	
C8	0.09812 (10)	0.7473 (3)	0.36750 (9)	0.0310 (4)	
H8	0.0879	0.6913	0.3211	0.037*	
C9	0.07341 (9)	0.9289 (3)	0.38248 (10)	0.0309 (4)	
H9	0.0457	0.9962	0.3469	0.037*	
C10	0.08921 (9)	1.0122 (3)	0.44958 (10)	0.0296 (4)	
H10	0.0734	1.1381	0.4596	0.036*	
C11	0.12842 (9)	0.9110 (3)	0.50231 (9)	0.0241 (3)	
H11	0.1391	0.9684	0.5483	0.029*	
C12	0.06366 (8)	0.5394 (3)	0.59313 (10)	0.0265 (4)	
H12A	0.0356	0.4433	0.6181	0.04*	
H12B	0.0424	0.5647	0.5447	0.04*	
H12C	0.0648	0.6579	0.6217	0.04*	
C13	0.19230 (8)	0.2421 (2)	0.67659 (9)	0.0184 (3)	
H13	0.2183	0.1852	0.6353	0.022*	
C14	0.23865 (9)	0.2287 (3)	0.74597 (9)	0.0239 (3)	
H14A	0.2823	0.3008	0.7395	0.036*	
H14B	0.2501	0.0944	0.756	0.036*	
H14C	0.2133	0.2823	0.787	0.036*	
C15	0.12439 (8)	0.1293 (2)	0.68651 (9)	0.0215 (3)	
H15A	0.0946	0.1382	0.642	0.032*	
H15B	0.099	0.1822	0.7276	0.032*	
H15C	0.136	−0.0051	0.6963	0.032*	
C16	0.24901 (8)	0.6727 (2)	0.73341 (8)	0.0146 (3)	
C17	0.19570 (8)	0.6985 (2)	0.78395 (8)	0.0153 (3)	
C18	0.21200 (7)	0.7788 (2)	0.85336 (8)	0.0157 (3)	
C19	0.28182 (8)	0.8304 (2)	0.86816 (8)	0.0164 (3)	
H19	0.2933	0.8853	0.9144	0.02*	
C20	0.33651 (7)	0.8063 (2)	0.81889 (8)	0.0152 (3)	
C21	0.31826 (7)	0.7257 (2)	0.75174 (8)	0.0154 (3)	
H21	0.3539	0.706	0.7173	0.018*	
O22	0.12784 (5)	0.64508 (18)	0.76713 (6)	0.0186 (2)	
C23	0.15478 (8)	0.8022 (2)	0.91039 (8)	0.0177 (3)	
C24	0.09529 (8)	0.9342 (3)	0.88101 (9)	0.0240 (3)	
H24A	0.0751	0.8813	0.8355	0.036*	
H24B	0.1145	1.0616	0.8717	0.036*	
H24C	0.0585	0.9437	0.9171	0.036*	
C25	0.12440 (9)	0.6043 (3)	0.92950 (9)	0.0220 (3)	
H25A	0.1051	0.5433	0.8851	0.033*	
H25B	0.0868	0.6198	0.9645	0.033*	
H25C	0.162	0.5241	0.9511	0.033*	
C26	0.18409 (9)	0.8921 (3)	0.98124 (9)	0.0224 (3)	
H26A	0.1461	0.9042	1.016	0.034*	
H26B	0.2033	1.019	0.9708	0.034*	
H26C	0.2216	0.8107	1.0022	0.034*	
C27	0.41164 (8)	0.8692 (2)	0.84125 (8)	0.0175 (3)	
C28	0.41262 (9)	1.0865 (3)	0.85531 (9)	0.0217 (3)	
H28A	0.3789	1.1178	0.8929	0.033*	
H28B	0.3995	1.1542	0.8101	0.033*	
H28C	0.4601	1.1257	0.8719	0.033*	
C29	0.46469 (8)	0.8238 (3)	0.78232 (9)	0.0241 (3)	
H29A	0.5118	0.8663	0.7987	0.036*	
H29B	0.4508	0.8905	0.7371	0.036*	
H29C	0.4654	0.6857	0.7735	0.036*	
C30	0.43590 (8)	0.7636 (3)	0.91139 (9)	0.0225 (3)	
H30A	0.4029	0.7905	0.9502	0.034*	
H30B	0.4832	0.8074	0.9266	0.034*	
H30C	0.437	0.6257	0.9021	0.034*	
H22	0.1327 (12)	0.561 (4)	0.7296 (12)	0.034 (6)*	

### Atomic displacement parameters (Å^2^)

**Table:**

	*U*^11^	*U*^22^	*U*^33^	*U*^12^	*U*^13^	*U*^23^
O1	0.0198 (5)	0.0170 (5)	0.0150 (5)	−0.0012 (4)	−0.0024 (4)	0.0005 (4)
C2	0.0140 (6)	0.0142 (7)	0.0170 (7)	−0.0007 (5)	−0.0014 (5)	−0.0003 (5)
N3	0.0157 (5)	0.0134 (6)	0.0175 (6)	−0.0004 (5)	−0.0026 (4)	0.0003 (5)
C4	0.0189 (7)	0.0196 (7)	0.0166 (7)	−0.0020 (6)	−0.0020 (5)	0.0000 (6)
C5	0.0178 (6)	0.0184 (7)	0.0143 (6)	−0.0009 (6)	−0.0006 (5)	−0.0022 (6)
C6	0.0172 (7)	0.0242 (8)	0.0157 (7)	−0.0047 (6)	0.0015 (5)	0.0016 (6)
C7	0.0264 (8)	0.0308 (9)	0.0172 (7)	−0.0099 (7)	0.0023 (6)	−0.0016 (7)
C8	0.0317 (9)	0.0466 (12)	0.0146 (7)	−0.0189 (9)	−0.0030 (6)	0.0049 (8)
C9	0.0228 (8)	0.0445 (12)	0.0249 (8)	−0.0102 (8)	−0.0044 (6)	0.0169 (8)
C10	0.0266 (8)	0.0332 (10)	0.0292 (9)	0.0026 (7)	0.0007 (7)	0.0102 (8)
C11	0.0258 (8)	0.0278 (9)	0.0185 (7)	0.0030 (7)	−0.0011 (6)	0.0021 (7)
C12	0.0162 (7)	0.0381 (10)	0.0249 (8)	−0.0015 (7)	−0.0024 (6)	0.0101 (8)
C13	0.0178 (7)	0.0148 (7)	0.0229 (7)	0.0015 (6)	0.0016 (5)	−0.0002 (6)
C14	0.0238 (8)	0.0186 (8)	0.0290 (8)	0.0014 (6)	−0.0048 (6)	0.0047 (7)
C15	0.0231 (7)	0.0167 (8)	0.0247 (8)	−0.0019 (6)	0.0026 (6)	0.0002 (6)
C16	0.0165 (7)	0.0122 (7)	0.0150 (6)	0.0010 (5)	−0.0006 (5)	0.0000 (5)
C17	0.0136 (6)	0.0138 (7)	0.0184 (7)	0.0010 (5)	−0.0006 (5)	0.0007 (5)
C18	0.0161 (6)	0.0138 (7)	0.0171 (7)	0.0018 (5)	0.0012 (5)	0.0012 (5)
C19	0.0171 (7)	0.0176 (7)	0.0145 (6)	0.0004 (5)	−0.0007 (5)	−0.0010 (6)
C20	0.0134 (6)	0.0149 (7)	0.0172 (7)	0.0003 (5)	−0.0005 (5)	0.0010 (6)
C21	0.0143 (6)	0.0153 (7)	0.0167 (6)	0.0014 (5)	0.0020 (5)	−0.0001 (6)
O22	0.0135 (5)	0.0222 (6)	0.0201 (5)	−0.0010 (4)	0.0000 (4)	−0.0036 (5)
C23	0.0146 (6)	0.0202 (8)	0.0183 (7)	0.0013 (6)	0.0023 (5)	−0.0004 (6)
C24	0.0206 (7)	0.0260 (9)	0.0256 (8)	0.0067 (7)	0.0036 (6)	0.0019 (7)
C25	0.0213 (7)	0.0246 (9)	0.0204 (7)	−0.0038 (6)	0.0029 (6)	0.0021 (6)
C26	0.0219 (7)	0.0259 (9)	0.0197 (7)	−0.0018 (7)	0.0041 (6)	−0.0045 (6)
C27	0.0135 (6)	0.0207 (8)	0.0181 (7)	−0.0012 (6)	−0.0004 (5)	−0.0014 (6)
C28	0.0200 (7)	0.0207 (8)	0.0243 (8)	−0.0034 (6)	−0.0002 (6)	−0.0030 (6)
C29	0.0141 (7)	0.0334 (9)	0.0248 (8)	−0.0021 (6)	0.0025 (6)	−0.0082 (7)
C30	0.0170 (7)	0.0271 (9)	0.0230 (7)	0.0024 (6)	−0.0030 (6)	0.0009 (7)

### Geometric parameters (Å, °)

**Table:**

O1—C2	1.4324 (18)	C16—C17	1.405 (2)
O1—C5	1.4345 (18)	C17—O22	1.3652 (17)
C2—N3	1.4947 (19)	C17—C18	1.416 (2)
C2—C16	1.512 (2)	C18—C19	1.390 (2)
C2—H2	1	C18—C23	1.539 (2)
N3—C13	1.485 (2)	C19—C20	1.407 (2)
N3—C4	1.4875 (19)	C19—H19	0.95
C4—C12	1.527 (2)	C20—C21	1.388 (2)
C4—C5	1.547 (2)	C20—C27	1.534 (2)
C4—H4	1	C21—H21	0.95
C5—C6	1.504 (2)	O22—H22	0.91 (3)
C5—H5	1	C23—C26	1.532 (2)
C6—C11	1.386 (3)	C23—C24	1.541 (2)
C6—C7	1.401 (2)	C23—C25	1.544 (2)
C7—C8	1.391 (3)	C24—H24A	0.98
C7—H7	0.95	C24—H24B	0.98
C8—C9	1.384 (3)	C24—H24C	0.98
C8—H8	0.95	C25—H25A	0.98
C9—C10	1.386 (3)	C25—H25B	0.98
C9—H9	0.95	C25—H25C	0.98
C10—C11	1.394 (2)	C26—H26A	0.98
C10—H10	0.95	C26—H26B	0.98
C11—H11	0.95	C26—H26C	0.98
C12—H12A	0.98	C27—C29	1.533 (2)
C12—H12B	0.98	C27—C30	1.541 (2)
C12—H12C	0.98	C27—C28	1.541 (2)
C13—C14	1.526 (2)	C28—H28A	0.98
C13—C15	1.526 (2)	C28—H28B	0.98
C13—H13	1	C28—H28C	0.98
C14—H14A	0.98	C29—H29A	0.98
C14—H14B	0.98	C29—H29B	0.98
C14—H14C	0.98	C29—H29C	0.98
C15—H15A	0.98	C30—H30A	0.98
C15—H15B	0.98	C30—H30B	0.98
C15—H15C	0.98	C30—H30C	0.98
C16—C21	1.395 (2)		
			
C2—O1—C5	103.35 (11)	C17—C16—C2	122.22 (13)
O1—C2—N3	104.08 (11)	O22—C17—C16	120.56 (13)
O1—C2—C16	110.87 (12)	O22—C17—C18	119.24 (13)
N3—C2—C16	114.02 (12)	C16—C17—C18	120.20 (13)
O1—C2—H2	109.2	C19—C18—C17	117.22 (13)
N3—C2—H2	109.2	C19—C18—C23	121.78 (13)
C16—C2—H2	109.2	C17—C18—C23	120.97 (13)
C13—N3—C4	113.28 (13)	C18—C19—C20	124.06 (14)
C13—N3—C2	115.16 (12)	C18—C19—H19	118
C4—N3—C2	106.19 (11)	C20—C19—H19	118
N3—C4—C12	110.11 (12)	C21—C20—C19	116.77 (13)
N3—C4—C5	102.71 (11)	C21—C20—C27	123.82 (13)
C12—C4—C5	113.85 (14)	C19—C20—C27	119.41 (13)
N3—C4—H4	110	C20—C21—C16	121.85 (13)
C12—C4—H4	110	C20—C21—H21	119.1
C5—C4—H4	110	C16—C21—H21	119.1
O1—C5—C6	112.09 (13)	C17—O22—H22	103.2 (14)
O1—C5—C4	102.43 (11)	C26—C23—C18	112.07 (12)
C6—C5—C4	114.71 (12)	C26—C23—C24	106.97 (14)
O1—C5—H5	109.1	C18—C23—C24	110.48 (12)
C6—C5—H5	109.1	C26—C23—C25	107.66 (13)
C4—C5—H5	109.1	C18—C23—C25	109.56 (13)
C11—C6—C7	119.14 (16)	C24—C23—C25	110.02 (13)
C11—C6—C5	122.20 (14)	C23—C24—H24A	109.5
C7—C6—C5	118.51 (16)	C23—C24—H24B	109.5
C8—C7—C6	119.79 (18)	H24A—C24—H24B	109.5
C8—C7—H7	120.1	C23—C24—H24C	109.5
C6—C7—H7	120.1	H24A—C24—H24C	109.5
C9—C8—C7	120.60 (17)	H24B—C24—H24C	109.5
C9—C8—H8	119.7	C23—C25—H25A	109.5
C7—C8—H8	119.7	C23—C25—H25B	109.5
C8—C9—C10	119.83 (17)	H25A—C25—H25B	109.5
C8—C9—H9	120.1	C23—C25—H25C	109.5
C10—C9—H9	120.1	H25A—C25—H25C	109.5
C9—C10—C11	119.83 (19)	H25B—C25—H25C	109.5
C9—C10—H10	120.1	C23—C26—H26A	109.5
C11—C10—H10	120.1	C23—C26—H26B	109.5
C6—C11—C10	120.75 (17)	H26A—C26—H26B	109.5
C6—C11—H11	119.6	C23—C26—H26C	109.5
C10—C11—H11	119.6	H26A—C26—H26C	109.5
C4—C12—H12A	109.5	H26B—C26—H26C	109.5
C4—C12—H12B	109.5	C29—C27—C20	112.13 (13)
H12A—C12—H12B	109.5	C29—C27—C30	107.72 (13)
C4—C12—H12C	109.5	C20—C27—C30	109.61 (13)
H12A—C12—H12C	109.5	C29—C27—C28	108.51 (14)
H12B—C12—H12C	109.5	C20—C27—C28	109.48 (13)
N3—C13—C14	111.70 (13)	C30—C27—C28	109.35 (14)
N3—C13—C15	109.08 (13)	C27—C28—H28A	109.5
C14—C13—C15	109.52 (13)	C27—C28—H28B	109.5
N3—C13—H13	108.8	H28A—C28—H28B	109.5
C14—C13—H13	108.8	C27—C28—H28C	109.5
C15—C13—H13	108.8	H28A—C28—H28C	109.5
C13—C14—H14A	109.5	H28B—C28—H28C	109.5
C13—C14—H14B	109.5	C27—C29—H29A	109.5
H14A—C14—H14B	109.5	C27—C29—H29B	109.5
C13—C14—H14C	109.5	H29A—C29—H29B	109.5
H14A—C14—H14C	109.5	C27—C29—H29C	109.5
H14B—C14—H14C	109.5	H29A—C29—H29C	109.5
C13—C15—H15A	109.5	H29B—C29—H29C	109.5
C13—C15—H15B	109.5	C27—C30—H30A	109.5
H15A—C15—H15B	109.5	C27—C30—H30B	109.5
C13—C15—H15C	109.5	H30A—C30—H30B	109.5
H15A—C15—H15C	109.5	C27—C30—H30C	109.5
H15B—C15—H15C	109.5	H30A—C30—H30C	109.5
C21—C16—C17	119.90 (13)	H30B—C30—H30C	109.5
C21—C16—C2	117.82 (13)		
			
C5—O1—C2—N3	−43.06 (13)	O1—C2—C16—C21	−92.31 (16)
C5—O1—C2—C16	−166.07 (12)	N3—C2—C16—C21	150.63 (13)
O1—C2—N3—C13	148.89 (12)	O1—C2—C16—C17	90.78 (16)
C16—C2—N3—C13	−90.19 (16)	N3—C2—C16—C17	−26.3 (2)
O1—C2—N3—C4	22.67 (15)	C21—C16—C17—O22	−179.16 (14)
C16—C2—N3—C4	143.59 (13)	C2—C16—C17—O22	−2.3 (2)
C13—N3—C4—C12	115.78 (16)	C21—C16—C17—C18	0.2 (2)
C2—N3—C4—C12	−116.86 (14)	C2—C16—C17—C18	177.09 (14)
C13—N3—C4—C5	−122.62 (14)	O22—C17—C18—C19	179.78 (14)
C2—N3—C4—C5	4.73 (15)	C16—C17—C18—C19	0.4 (2)
C2—O1—C5—C6	169.17 (12)	O22—C17—C18—C23	1.4 (2)
C2—O1—C5—C4	45.70 (14)	C16—C17—C18—C23	−178.02 (14)
N3—C4—C5—O1	−30.41 (15)	C17—C18—C19—C20	−0.5 (2)
C12—C4—C5—O1	88.60 (15)	C23—C18—C19—C20	177.89 (15)
N3—C4—C5—C6	−152.11 (13)	C18—C19—C20—C21	0.0 (2)
C12—C4—C5—C6	−33.09 (19)	C18—C19—C20—C27	179.87 (15)
O1—C5—C6—C11	−20.2 (2)	C19—C20—C21—C16	0.7 (2)
C4—C5—C6—C11	96.10 (18)	C27—C20—C21—C16	−179.21 (15)
O1—C5—C6—C7	164.23 (13)	C17—C16—C21—C20	−0.8 (2)
C4—C5—C6—C7	−79.51 (18)	C2—C16—C21—C20	−177.78 (14)
C11—C6—C7—C8	−2.4 (2)	C19—C18—C23—C26	1.8 (2)
C5—C6—C7—C8	173.36 (14)	C17—C18—C23—C26	−179.90 (15)
C6—C7—C8—C9	0.9 (2)	C19—C18—C23—C24	120.96 (16)
C7—C8—C9—C10	1.1 (3)	C17—C18—C23—C24	−60.72 (19)
C8—C9—C10—C11	−1.6 (3)	C19—C18—C23—C25	−117.67 (16)
C7—C6—C11—C10	1.9 (2)	C17—C18—C23—C25	60.66 (18)
C5—C6—C11—C10	−173.66 (16)	C21—C20—C27—C29	−2.7 (2)
C9—C10—C11—C6	0.1 (3)	C19—C20—C27—C29	177.39 (15)
C4—N3—C13—C14	170.85 (12)	C21—C20—C27—C30	−122.31 (16)
C2—N3—C13—C14	48.35 (18)	C19—C20—C27—C30	57.81 (19)
C4—N3—C13—C15	−67.93 (16)	C21—C20—C27—C28	117.75 (17)
C2—N3—C13—C15	169.56 (12)	C19—C20—C27—C28	−62.13 (19)

### Hydrogen-bond geometry (Å, °)

**Table:**

*D*—H···*A*	*D*—H	H···*A*	*D*···*A*	*D*—H···*A*
O22—H22···N3	0.91 (3)	1.75 (2)	2.6180 (17)	158 (2)
